# CD26 is a senescence marker associated with reduced immunopotency of human adipose tissue-derived multipotent mesenchymal stromal cells

**DOI:** 10.1186/s13287-022-03026-4

**Published:** 2022-07-26

**Authors:** Rose Triantafillia Psaroudis, Urvashi Singh, Maximilien Lora, Peter Jeon, Abigail Boursiquot, Ursula Stochaj, David Langlais, Inés Colmegna

**Affiliations:** 1 Research Institute of the McGill University Health Centre, 1001 Decarie Blvd, Office # EM2-3238, Montreal, QC H4A 3J1 Canada; 2 Department of Physiology, Faculty of Medicine and Health Sciences, McGill University, Montreal, QC Canada; 3 Department of Human Genetics, Faculty of Medicine and Health Sciences, McGill University, Montreal, QC Canada; 4 Department of Microbiology and Immunology, Faculty of Medicine and Health Sciences, McGill University, Montreal, QC Canada; 5 Division of Rheumatology, Department of Medicine, Faculty of Medicine, McGill University, Montreal, QC Canada

**Keywords:** Mesenchymal stromal cells (MSCs), Adipose tissue (AT), CD26, Dipeptidyl peptidase 4 (DPP4), Senescence, Aging

## Abstract

**Introduction:**

Human mesenchymal stromal cells (MSCs) have immunomodulatory, anti-inflammatory, and tolerogenic effects. Long-term in vitro expansion of MSCs to generate clinical grade products results in the accumulation of senescent-functionally impaired MSCs. Markers to assess the ‘senescent load’ of MSC products are needed.

**Methods:**

Early and late passage human adipose tissue (AT) MSCs from pediatric and adult donors were characterized using established senescent markers [i.e., MSC size, granularity, and autofluorescence by flow cytometry; β-galactosidase staining (SA-β-gal); *CDKN2A* and *CDKN1A* by qRT-PCR]. In gene set enrichment analysis, DPP4 (also known as adenosine deaminase complexing protein 2 or CD26) was found as a prominent dysregulated transcript that was increased in late passage MSC(AT). This was confirmed in a larger number of MSC samples by PCR, flow cytometry, Western blotting, and immunofluorescence. In vitro immunopotency assays compared the function of CD26^high^ and CD26^low^ MSC(AT). The effect of senolytics on the CD26^high^ subpopulation was evaluated in senescent MSC(AT).

**Results:**

Late passage MSC(AT) had a senescence transcriptome signature. *DPP4* was the most differentially enriched gene in senescent MSCs. Late passage senescent MSC(AT) had higher CD26 surface levels and total protein abundance. Moreover, CD26 surface levels were higher in early passage MSC(AT) from adults compared to pediatric donors. CD26 abundance correlated with established senescence markers. CD26^high^ MSC(AT) had reduced immunopotency compared to CD26^low^ MSC(AT). Senolytic treatment induced MSC apoptosis, which decreased the frequencies of CD26^high^ MSC(AT).

**Conclusions:**

*DPP4* gene expression and DPP4/CD26 protein abundance are markers of replicative senescence in MSC(AT). Samples enriched in CD26^high^ MSC(AT) have reduced immunopotency and CD26^high^ MSCs are reduced with senolytics.

**Supplementary Information:**

The online version contains supplementary material available at 10.1186/s13287-022-03026-4.

## Introduction

Mesenchymal stromal cells (MSCs) are a heterogeneous population of multipotent adult progenitor cells with unique phenotypic features [1]. Functionally, MSCs display broad immunomodulatory, pro-angiogenic, and anti-fibrotic effects [2]. This provides a rationale for current clinical trials testing the therapeutic potential of MSCs. MSCs can be derived from several tissue sources, most commonly bone marrow [MSC(M)], adipose tissue [MSC(AT)], and Wharton’s jelly [MSC(WJ)] [3]. A prerequisite for successful MSC-based therapies is a large-scale manufacturing process that preserves the biological potency of the initial MSC population [4]. However, the expansion of MSCs has the intrinsic risk that cells undergo replicative senescence.

Cellular senescence is a physiological state that can be triggered by stressful insults and other processes. It is characterized by a prolonged and generally irreversible cell cycle arrest with secretory features [senescent-associated secretory phenotype (SASP)], macromolecular damage, and altered metabolism [5]. Senescent MSCs are functionally impaired (e.g., decreased immunomodulatory capacity) and can contribute to age-related diseases [6]. Furthermore, a clinical product with high levels of senescent MSCs may have reduced therapeutic effects [7]. Currently, there are no markers with high specificity for senescent MSCs, and clinically validated senescence-biomarkers are not available [8]. The lack of reliable senescence markers limits the ability to determine the ‘senescent load’ in MSC-based products.

The goal of this study was to identify functionally relevant senescent markers in MSC(AT). To accomplish this, we performed gene set enrichment analysis to compare the expression of senescent transcripts in early passage (EP, non-senescent) and late passage (LP, senescent) human MSC(AT). We identified *DPP4*, which encodes dipeptidyl peptidase-4 (also called CD26 protein), as one of the most differentially expressed genes. The differential expression and surface abundance of CD26 was confirmed in a larger number of early and late passage MSCs as well as in MSCs from pediatric and adult donors. MSCs enriched for CD26 were characterized by reduced immunopotency. Notably, senolytic treatment removed CD26^high^ MSCs.

## Material and methods

### Study subjects

The McGill University Health Centre Ethics Review Board approved the study and all participants provided written informed consent (10-107GEN). Subcutaneous adipose tissue (AT) was obtained from seven adults undergoing programmed coronary artery bypass graft surgery and seven children undergoing elective orthopedic procedures. Additional file 1: Table S1 depicts the demographic information of the study participants.

### MSC(AT) isolation and culture

Human MSCs were isolated from AT as previously described [9, 10]. Briefly, subcutaneous AT samples were washed with phosphate buffered saline (PBS) (Wisent Inc., St. Bruno, QC) and minced with surgical scissors. The tissue was digested with 0.05% collagenase (Sigma‐Aldrich Corporation, MO, USA) dissolved in Hank’s balanced salt solution (Invitrogen, MA, USA). After 2 h at 37C, the collagenase was neutralized with the addition of fetal bovine serum (FBS), and samples were centrifuged (5 min at 2000 rpm). The pellet was resuspended in complete low glucose Dulbecco’s modified Eagle’s medium (DMEM) (Wisent Inc., St. Bruno, QC), supplemented with 10% MSC-qualified FBS (certified FBS-MSC, Gibco Invitrogen) and 1% penicillin/streptomycin (Wisent Inc., St. Bruno, QC). Digested tissue samples were cultured in T75 flasks at 37 °C in 5% CO_2_ (1 g of tissue/flask), and non-adherent cells were washed off two days following isolation. Isolated MSC(AT) were seeded at a density of 4,000 cells/cm^2^ in T75 flasks in complete medium and passaged at 80% confluency. Early and late passage MSC(AT) were defined as ≤ 5 and ≥ 15 passages respectively [11, 12].

### MSC(AT) characterization

MSC(AT) immunophenotyping was performed with multiparametric flow cytometry analysis [BD Biosciences, LSRFortessa cell analyzer] as previously described [10, 13]. Data were analyzed using FlowJo software version 9.9.6 (BD Life Sciences, USA). Tri-lineage differentiation of MSC(AT) was tested with the StemPro Adipogenesis, Osteogenesis, Chondrogenesis Differentiation Kit (Waltham, MA) according to the manufacturer’s protocol. Chondrocyte pellets were mounted, sectioned, and stained with Alcian Blue.

### Evaluation of senescence markers

#### Senescence associated β-galactosidase (SA-β-gal)

SA-β-gal staining was performed according to the manufacturer’s instructions (Cell Signaling Technology, Danvers, MA). MSC(AT) were co-stained with DAPI (4′,6-diamidino-2-phenylindole) for quantification. Images were acquired with a Olympus IX51 microscope, and the ratio of SA-β-gal^pos^/DAPI^pos^ was determined with Image J for > 100 MSCs per sample.

#### Flow cytometry

Cell size (forward scatter area [FSC-A]), granularity (side scatter area [SSC-A]), autofluorescence (Alexa Fluor 488 channel), and CD26 surface abundance [allophycocyanin (APC)‐conjugated monoclonal anti-CD26 (Biolegend, San Diego, CA)] were assessed by flow cytometry [BD Biosciences, LSRFortessa cell analyzer]. Data were analyzed with FlowJo software version 9.9.6.

#### Quantitative reverse transcription polymerase chain reaction (qRT-PCR)

This was performed in MSCs previously characterized for senescence markers. Total RNA was extracted from 100K MSC(AT) with TRIzol (Ambion Life Technologies, CA, USA). RNA was purified with the Direct-zol RNA MiniPrep Kit (Zymo Research, CA, USA). Complementary DNA (cDNA) was synthesized with the QuantiTect reverse transcription kit (Qiagen, Germany), and PCR was conducted with the QuantiNova SYBR Green PCR kit (Qiagen, Germany) according to the manufacturer’s protocol. Gene primer sequences are listed in Additional file 1: Table S3. Relative expression of Gene of Interest/*HPRT* was used to compare the mRNA levels in EP- and LP-MSC(AT).

### RNA-Seq and analysis

#### Sequencing

EP- and LP-MSC(AT) from a pediatric and an adult donor were tested. MSC(AT) were preserved in RNAprotect Cell Reagent (Qiagen, Germany) and stored at − 80 °C until RNA isolation. RNA was isolated with the RNeasy Micro Kit (Qiagen, Germany) according to the manufacturer’s protocol. RNA library preparation and sequencing were done by the molecular biology platform of the Institut de recherches cliniques de Montréal (IRCM). The RNA was quantified with a Qubit (Invitrogen), and RNA quality was measured on a BioAnalyzer chip (Agilent); the RNA integrity (RIN) was 7 or greater. Ribosomal RNA was depleted for 400 ng of total RNA (Ribocop depletion kit v 3.1; Lexogen), and libraries were prepared with the KAPA RNA hyperprep kit (Roche). Briefly, depleted RNA was reverse transcribed, the resulting cDNA was fragmented (94 °C 8 min), blunt-ended, and indexed adaptors were ligated. Ligated products were then amplified by PCR (9 cycles) and purified on KAPA pure beads (1:1 ratio). The final library had an average size distribution of 344 bp, and libraries were quantified by qPCR. Libraries were then paired-end sequenced (50 bp) on the Illumina Novaseq 6000 (Illumina) with S1 flowcell and an average coverage of 60–80 M fragments per library.

#### Analysis

The quality of sequence reads was assessed with the FastQC tool (Babraham Bioinformatics). Trim of low-quality bases and removal of sequencing adapters was done with Trimmomatic v.0.36 [14]. FastQC confirmed the high quality of the trimmed sequence reads, which were then mapped to the human UCSC GRCh38 reference assembly using HISAT2 v2.2.0 with parameters -k 1 to avoid multi-mapped reads,—rna-strandness FR for strand-specific information, and—trim5 6 to trim 6 bases from the 5′ end of each read before alignment [15]. Samtools converted the files from the SAM to BAM format; it also sorted and indexed these files [16]. Gene expression was quantified by counting the number of uniquely mapped reads using featureCounts with the -s parameter to perform strand-specific read counting [17]. Reads mapped to rRNAs were removed to resolve any biases from incomplete ribo-depletion. Genes with a minimum expression level of five counts per million (CPM) reads in at least 2 of the samples were retained. Genes with sex-specific expression (XIST and ChrY genes) were excluded to reduce donor variability. TMM normalization was applied with the edgeR Bioconductor package, and EP- and LP- samples were compared by pairwise differential gene expression analysis [18]. Normalized gene expression (counts per million; cpm) was submitted to gene set enrichment analysis (GSEA) [19], using a senescence-associated gene set created from published data (Additional file 1: Table S2). Plots were generated with the ggplot2 package.

### CD26^+^ MSC(AT)

#### Western blot analysis (WB)

The preparation of crude MSC(AT) extracts and WB was previously described [20]. The following primary antibodies were used at the dilutions indicated: CD26 (OriGene Technologies Inc., MD, USA; diluted 1:500), β-actin (Chemicon MAB1501; diluted 1: 100,000). HRP-coupled secondary antibodies (Jackson, ImmunoResearch Laboratories, USA) were diluted 1:2,000. Enhanced chemiluminescence (ECL) signals were acquired with a ChemiDoc™ (BioRad), quantified, and normalized to β-actin intensities.

#### Confocal microscopy

MSC(AT) were fixed and processed for immunofluorescence as described [21]. Images were acquired with a Zeiss LSM780 in the multi-track mode, using a 63Χ oil immersion objective (NA 1.4). Images were processed with Adobe Photoshop CS4.

#### Fluorescence-activated cell sorting (FACS)

CD26^low^ and CD26^high^ populations were sorted with a BD FACSAria Fusion sorter (BD Biosciences) and collected in 100% FBS (Gibco Invitrogen). Sorted MSC(AT) were cultured in complete medium for 48 h prior to assessing their immunopotency.

### Immunopotency assay

The MSC(AT) ability to suppress activated CD4^+^ T cell proliferation was evaluated in an allogenic co-culture system as previously described [10]. Briefly, FACS-sorted CD26^low^ and CD26^high^ MSC(AT) were grown in 96-well plates (12,500 cells/well) and cultured overnight. Monocyte-depleted peripheral blood mononuclear cells (PBMCs) stained with carboxyfluorescein succinimidyl ester (CFSE) (Sigma-Aldrich, Oakville, ON) and activated with Dynabeads™ Human T-Activator CD3/CD28 beads (Thermo Fisher Scientific, Burlington, ON) were co-cultured for 72 h with the MSC(AT) at a MSC: PBMC ratio of 1:16. PBMCs were labeled with anti-CD4 monoclonal antibodies (APC-conjugate, # 555349, BD Biosciences, San Diego, CA) and the viability marker 7‐aminoactinomycin D (7-AAD) (# 559925, BD Biosciences, San Diego, CA) and analyzed by flow cytometry. The percentage of CD4^+^ T cell proliferation was determined with the Proliferation Platform of FlowJo software (version 9.9.6).

### Treatment with senolytics

LP-MSC(AT) were seeded at a density of 50,000 cells/well in 6-well plates. Samples were untreated (control), incubated with dimethyl sulfoxide (DMSO) (vehicle control) or with a senolytic at concentrations previously optimized for MSC(AT). Senolytics were present for 48 h at the following final concentrations: dasatinib (64 μM), navitoclax (20 μM), quercetin (400 μM and 800 μM) or 17-DMAG (25.6 μM and 51.2 μM). Following treatment, MSC(AT) were labeled with CD26, DRAQ7 (cell impermeable DNA stain, Abcam, Cambridge, MA), and Annexin V (BD Biosciences, San Diego, CA). Samples were analyzed by flow cytometry.

### Statistical analysis

Analyses were performed with GraphPad Prism software version 8.3.0 (GraphPad, CA, USA). Two-tailed Student’s *t*-test (WB), paired one-way ANOVA (senolytics treatment), paired Wilcoxon tests [EP- versus LP-MSC(AT) comparisons], unpaired and nonparametric Mann–Whitney test (pediatric versus adult comparisons) were used. Simple linear regressions were used to correlate CD26 surface abundance with other markers of cell senescence. Means ± standard deviation (SD) for all comparisons were reported. Significance was defined as *p < 0.05, **p < 0.01, ***p < 0.001, and ****p < 0.0001.

## Results

### LP-MSC(AT) are senescent

MSC(AT) were characterized according to the minimal criteria of the International Society for Cell and Gene Therapy (ISCT) [1]. EP- and LP-MSC(AT) adhered to plastic, were positive for CD73, CD90 and CD105, and negative for HLA-DR and hematopoietic surface markers (Additional file 1: Fig S1A–B). In addition, all MSC(AT) showed trilineage differentiation capacity (i.e., adipocytes, chondroblasts, and osteoblasts) when exposed to the appropriate growth conditions in vitro (Additional file 1: Fig S1C). Applying these criteria, all MSC(AT) tested in the current study conformed to the ISCT minimal standards.

**Fig. 1:**
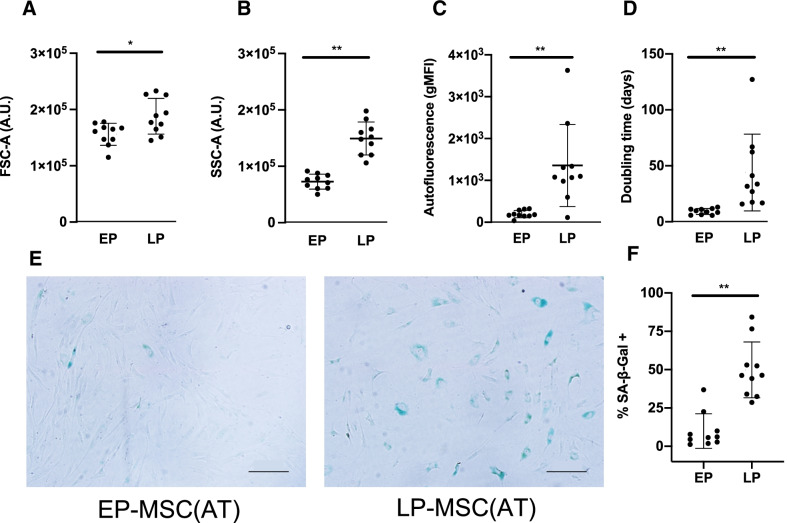
Late passage MSC(AT) are enriched in senescence markers. **A** Size (FSC-A), **B** granularity (SSC-A), **C** autofluorescence, **D** doubling time (days), **E** representative example of β-galactosidase staining (SA-β-Gal), and **F** summary data for percent of SA-β-Gal in early passage (EP) and late passage (LP) MSC(AT). Graphs **A**–**D** and** F** summarize data of 6 adult and 4 pediatric MSC(AT) samples at EP (p4.1 ± 0.6) and LP (p21.0 ± 6.0). Data shown as mean ± SD, comparisons done using paired Wilcoxon test, *p < 0.05, **p < 0.01. Scale bars in **E** represent 200 μm

To further characterize the phenotype of LP-MSC(AT), we evaluated their size (FSC-A), granularity (SSC-A), autofluorescence, SA-β-gal activity, and doubling time (Additional file 1: Fig S2). LP-MSC(AT) were larger, more granular, and with higher autofluorescence than EP-MSC(AT) (Fig. 1A–C). The doubling time and the percentage of SA-β-gal positive cells were also increased for LP-MSC(AT) (Fig. 1D–F). Overall, these data indicate that LP-MSC(AT) display a senescent phenotype.

### LP-MSC(AT) have a senescent transcriptome

**Fig. 2:**
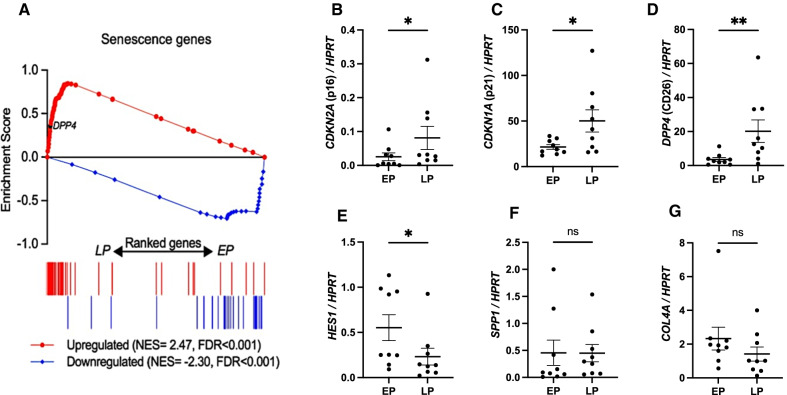
Late passage (LP) MSC(AT) have a senescent transcriptome signature. **A** Gene set enrichment analysis demonstrates increased expression of genes known to be upregulated in senescence (shown in red) and reduced expression of genes downregulated in senescence (shown in blue) in LP-MSC(AT) and shows *DPP4* among core enrichment genes; *n* = 1 pediatric and *n* = 1 adult, where EP = p6 and LP = p30. **B**–**G** Relative expression of selected transcripts from RNAseq analysis tested by quantitative RT-PCR over HPRT housekeeping gene in 9 additional samples. Expression of **B** *CDKN2A* (p16), **C** *CDKN1A* (p21), **D** *DPP4 (*CD26), **E** *HES1*,** F** *SPP1* and **G** *COL4A1* over the house-keeping gene *HPRT*. Data presented as mean ± SD, comparisons done with paired Wilcoxon test, *p < 0.05, **p < 0.01

Next we assessed if LP-MSC(AT) had a senescent transcriptomic profile. We conducted an exploratory RNA-seq on EP- and LP-MSC(AT) from one pediatric and one adult donor. We performed gene set enrichment analysis (GSEA) using two sets of genes generated based on previous reports (i.e., genes that are (1) upregulated or (2) downregulated during cellular senescence) [22–26] (Additional file 1: Table S2). GSEA confirmed a global senescence signature in the LP-MSC(AT) samples (Fig. 2A). *DPP4 (CD26)* was among the core enrichment genes (Fig. 2A). EP-MSC(AT) were enriched for genes downregulated in senescence (Fig. 2A). Among the upregulated genes, forkhead box E1 (*FOXE1)* and secreted phosphoprotein 1 (*SPP1)* were previously reported to be higher expressed in senescent human MSCs [22]. FOXE1 is a transcription factor with poorly defined function in senescence, whereas *SPP1* encodes osteopontin, a secreted stromal protein involved in tumor growth [27].

To validate the results of the RNA-seq analysis, we tested the expression of *DPP4, FOXE1, SPP1,* collagen type IV alpha 1 (*COL4A1)* and hairy and enhancer of split-1 (*HES1)* in nine additional EP- and LP-MSC(AT). We included *CDKN2A* and *CDKN1A* as prototype genes overexpressed in senescent MSC(AT) and *HPRT* as a housekeeping gene. We confirmed the overexpression of both *CDKN2A* (Fig. 2B) and *CDKN1A* (Fig. 2C) in the LP-MSC(AT). In addition, *DPP4* expression was significantly higher in LP-MSC(AT) (Fig. 2D), while *HES1* was downregulated at late passage (Fig. 2E). The expression of *SPP1* (Fig. 2F) and *COL4A1* (Fig. 2G) followed the expected differences but did not reach significance. *FOXE1* was not detected in all samples and had high cycle threshold values (Ct), indicating that it is not a suitable senescence marker for MSC(AT) (data not shown). Overall, these results confirm that the LP-MSC(AT) transcriptome have a senescent signature. The data highlight the potential of *CD26* transcript abundance to serve as a senescence marker.

### CD26 protein levels are elevated in LP-MSC(AT) and MSCs from adult donors

**Fig. 3:**
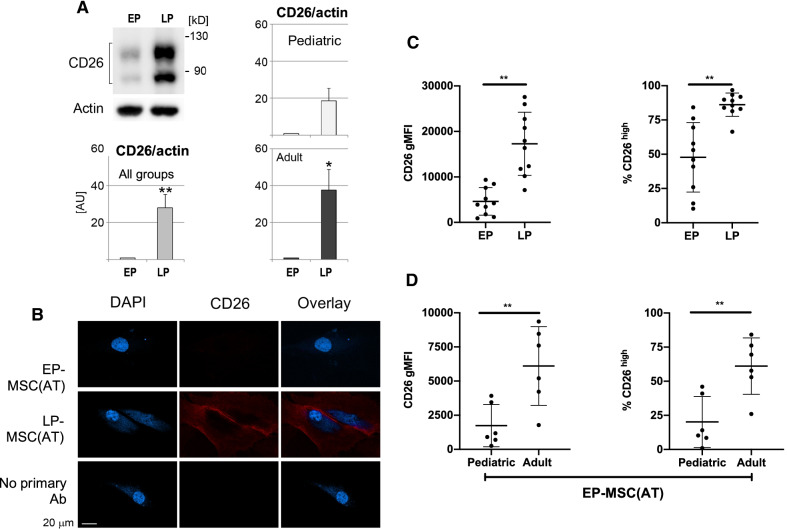
CD26 surface levels and total protein abundance in MSC(AT) increase with replicative senescence and age. **A** Western Blot of CD26 protein levels in MSC(AT) extracts from early passage (EP) and late passage (LP) pediatric (*n* = 2) and adult (*n* = 3) samples. Data represent mean + SEM, comparisons done with two-tailed Student’s *t*-test, *p < 0.05; **p < 0.01. **B** Representative immunohistochemistry image of CD26 protein abundance in EP and LP-MSC(AT). **C** CD26 geometric mean fluorescence intensity (gMFI) and % CD26^high^ MSCs in EP and LP-MSC(AT) (6 adult and 4 pediatric MSC(AT), at EP = p4.1 ± 0.6 and LP = p21.0 ± 6.0). Data represent mean ± SD, comparisons done with paired Wilcoxon tests, **p < 0.01. **D** CD26 gMFI and % CD26^high^ MSC(AT) in EP-MSC(AT) from pediatric and adult donors (6 pediatric MSC(AT) at *p* = 4.7 ± 0.5 and 6 adult MSC(AT) *p* = 3.8 ± 0.4). Data represent mean ± SD, comparisons done with unpaired Mann–Whitney tests, **p < 0.01

Western blot analysis determined whether the increase of *CD26* gene expression leads to changes in protein abundance. Two bands of ~ 110kD and ~ 85kD molecular mass were detected for CD26 (Fig. 3A). The upper band observed in the WB is likely the full length CD26/DPP4 protein, and the lower band likely corresponds to the soluble form of CD26/DPP4. Both bands were present in MSC(AT) from different donors at all passages. However, the abundance of CD26 protein markedly increased when MSCs were cultured to late passage (Fig. 3A). While the same trend was observed for MSCs from pediatric and adult donors, the changes were more pronounced for adult donors.

As CD26 resides in the plasma membrane of fibroblasts [28], we located the protein in MSC(AT) by indirect immunofluorescence combined with confocal microscopy (Fig. 3B, Additional file 1: Fig S3). Consistent with results for Western blotting, fluorescence signals were low at early passage, but profoundly elevated at late passage. High levels of CD26 were detected in the cytosol and at the cell periphery of LP-MSC(AT) (Fig. 3B). The accumulation of CD26 at the cell periphery is consistent with the plasma membrane localization of the protein.

The surface localization and abundance of CD26 was independently confirmed by flow cytometry (Additional file 1: Fig S4). Compared with EP-MSC(AT), in LP-MSC(AT) the CD26 surface staining was increased and the CD26^high^ subpopulation was larger (Fig. 3C). Notably, the levels of CD26 at the MSC(AT) surface positively correlated with cell granularity, SA-β-gal activity and to a lesser extent with autofluorescence (Additional file 1: Fig S5).

Next, we evaluated whether the levels of cell surface CD26 are suitable to discriminate between early passage MSC(AT) from pediatric and adult donors. CD26 surface protein was increased in EP-MSC(AT) from adult compared to pediatric donors (Fig. 3D). In addition, samples from adult donors had a significantly higher percentage of CD26^high^ MSC(AT) (Fig. 3D). Together, these data support the idea that CD26 provides a marker of MSC(AT) replicative senescence. CD26 abundance may also provide a biomarker to distinguish MSCs from donors of different biological age groups.

### CD26^high^ MSC(AT) have reduced immunosuppressive capacity

**Fig. 4:**
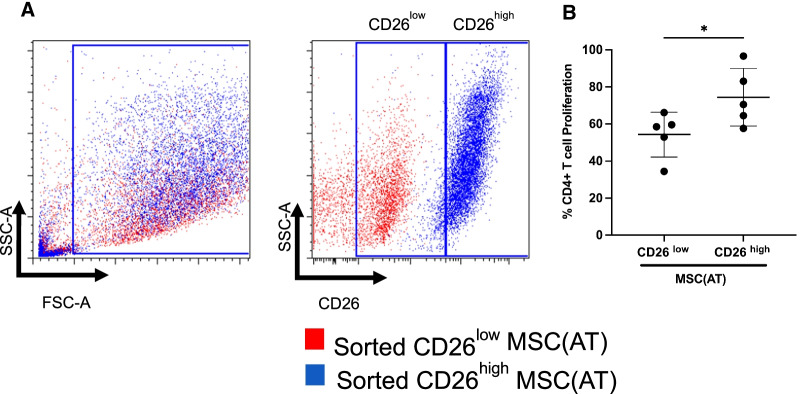
CD26^high^ MSC(AT) are less immunosuppressive than CD26^low^ MSC(AT).** A** Flow cytometry gating strategy for FACS separation of MSC(AT) based on CD26 surface abundance: CD26^low^ and CD26^high^ populations. **B** Immunopotency assay (i.e., MSC inhibition of proliferating CD4^+^ T cells). CD3CD28 activated T-cells were co-cultured with either CD26^low^ or CD26^high^ MSC(AT) at a 1:16 MSC:PBMC ratio. *n* = 4 adult and 1 pediatric MSC(AT). Data represent mean ± SD, comparisons done with paired Wilcoxon tests, *p < 0.05

Senescent MSCs have impaired immunomodulatory properties [29]. To evaluate a possible link between immunomodulation and CD26 surface levels, MSC(AT) were FACS sorted into CD26^low^ and CD26^high^ subpopulations (Fig. 4A). MSC:T-cell suppression assays were then performed with each subpopulations. CD26^high^ MSC(AT) were less potent to suppress CD4 T-cell proliferation than CD26^low^ MSC(AT) (Fig. 4B). These results highlight the reduced immunosuppressive functions of senescent CD26^high^ MSC(AT).

### Senolytics reduce the percentage of CD26^high^ MSC(AT)

**Fig. 5:**
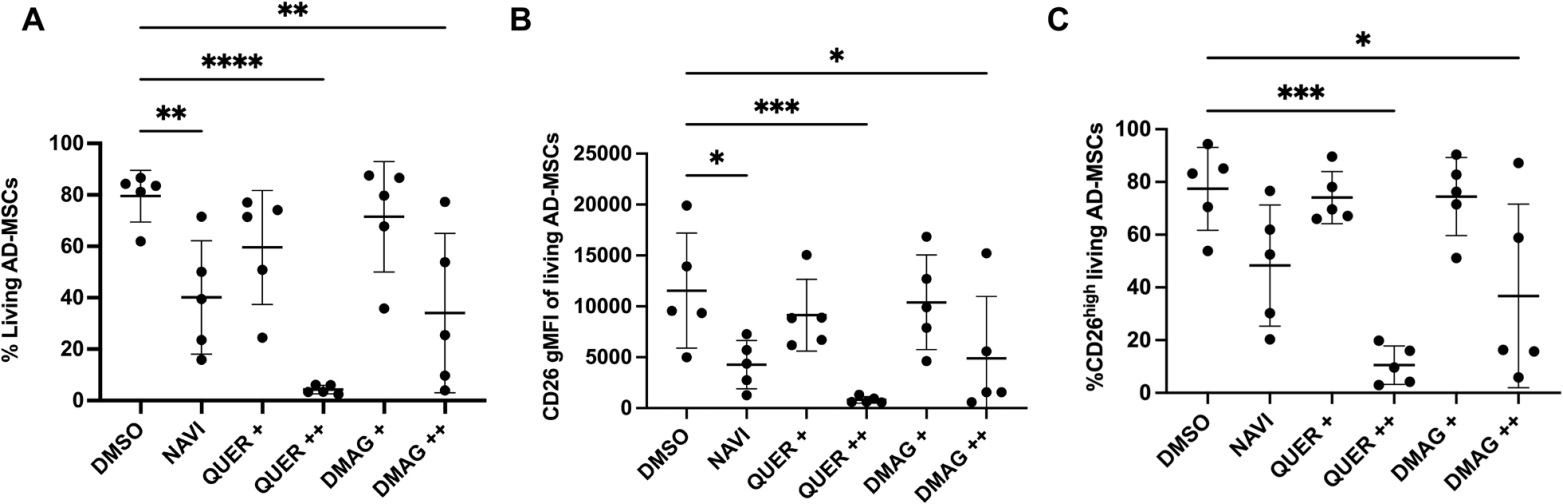
Viability and CD26 surface levels in late passage MSC(AT) decrease following senolytic treatment. **A** Cell viability of late passage MSC(AT) that were either treated with DMSO (vehicle control) or a senolytic: NAVI: navitoclax (20 μM), QUER: quercetin (+:400 and ++:800 μM) and DMAG: 17-DMAG (+:25.6 and ++:51.2 μM). **B** CD26 gMFI and **C** %CD26^high^ MSCs among living MSC(AT). [*n* = 4 adult and 1 pediatric LP-MSC(AT)]. Data presented as mean ± SD, comparisons done with paired one-way ANOVA, *p < 0.05, **p < 0.01, ***p < 0.001, ****p < 0.0001

Senolytic agents can eliminate senescent cells from mixed cultures or aging organisms [30]. This prompted us to assess the effect of senolytics on the abundance of CD26^high^ MSC(AT). Senescent MSC(AT) were treated with DMSO (vehicle control) or different senolytic compounds at concentrations previously optimized (data not shown). Subsequently, MSC(AT) cell viability and CD26 surface levels were measured by flow cytometry. While DMSO did not induce MSC(AT) death, all senolytics diminished MSC(AT) viability (Fig. 5A). Moreover, navitoclax and high doses of quercetin and 17-DMAG reduced the percentage of CD26^high^ MSCs as well as the surface abundance of the CD26 protein (Fig. 5B–C). These results suggest that senolytic treatment can reduce the number or remove senescent CD26^high^ MSC(AT).

## Discussion

MSCs are ideal candidates for cell therapies; they can be easily isolated from various tissue sources, have sizeable proliferation potential, and potent immunomodulatory and anti-fibrotic properties [2]. However, the extensive in vitro expansion of MSCs needed to obtain clinical grade products leads to the accumulation of senescent dysfunctional MSCs. There is currently no universal feature that identifies a senescent cell. Therefore, simultaneously testing several biomarkers is required to define the senescent state [31]. In this study, we first characterized EP/LP MSC(AT) by determining SA-β-gal activity, the expression of cyclin-dependent kinase inhibitors (*CDKN2A*, *CDKN1A*), MSC size, granularity, autofluorescence and doubling time. Furthermore, we interrogated the transcriptome of EP/LP MSC(AT) for senescence markers identifying CD26 as candidate. Our study demonstrated that the increase in *CD26* expression and protein abundance correlates with MSC senescence. Importantly, we show that CD26^high^ MSC(AT) have reduced immunopotency compared with CD26^low^. Cellular senescence is a driver of aging. Therefore, we quantified CD26^high^ MSC(AT) in cultures established from adult and pediatric donors. Consistent with aging-associated changes, the percentage of CD26^high^ MSC(AT) is elevated for adult donors. Finally, we developed protocols to eliminate CD26^high^ MSC(AT) from mixed cultures by treatment with senolytics.

The ubiquitous protease CD26 cleaves amino-terminal dipeptides from various substrates [32, 33]. As such, CD26 regulates critical biological processes related to metabolism, tissue regeneration, and immune responses [34]. CD26 levels are elevated in diabetes and CD26 inhibitors are used as hypoglycemic agents. The beneficial effect of CD26 inhibitors exceeds their effects on metabolism extending to pro-angiogenic and anti-fibrotic effects [34].

Senescent cells accumulate during aging and promote a variety of age-associated diseases [31]. The link between CD26 and senescence was first reported in fibroblasts where DPP4 enhances the growth suppression characteristic of senescence [28]. DPP4 silencing decreased the production of reactive oxygen species in pre-senescent fibroblasts and led to reductions in p53, p21 and p16 and to the increase in SIRT1. Recently, the expression of CD26 in senescent umbilical cord blood MSCs was described [35]. In that study, sorted CD26^+^ MSC grew slower than CD26^−^ MSC, had increased SA-β-gal, diminished expression of Oct4 and Nanog and were characterized by a SASP. Pharmacologic inhibition or knockdown of CD26 delayed MSCs replicative senescence and, in a mouse model of emphysema, was associated with higher therapeutic efficacy [35]. In the current study we show that early passage adult MSC(AT) have increased CD26 surface abundance and a higher percentage of CD26^high^ cells compared to pediatric MSC(AT). This highlights the biological relevance of CD26 surface protein abundance; it is not only increased during replicative senescence but also during biological aging. It should be noted, however, that CD26 abundance does not increase with age in all cell types (i.e., CD26 is not a universal indicator of cellular senescence). For example, CD26 levels diminish in CD8 T-cells with age, while no age-dependent changes occurred in CD4 T-cells [36]. Collectively, our data emphasize the importance of donor age when selecting MSCs for therapeutic use [37].

Senescent cells often elevate negative regulators of apoptosis, which may confer resistance to apoptosis-inducing signals [31]. Senolytics may target these anti-apoptotic pathways and eliminate senescent cells. To achieve this, we optimized the MSC(AT) treatment with four senolytics (dasatinib, navitoclax, quercetin, and 17-DMAG) that affect different cellular pathways. Navitoclax (Bcl-2 inhibitor), high doses of quercetin (PI3K inhibitor), and 17-DMAG (HSP90 inhibitor) increased MSC(AT) apoptosis and reduced the total CD26 surface abundance in living MSC(AT). This removal of CD26^high^ with senolytics supports the concept that CD26 is a senescence marker in MSC(AT).

A limitation of this study is that we analyzed the transcriptome of only two EP and two LP human MSC(AT) samples. Therefore, our RNA-seq results could serve for hypothesis generation but do not allow concluding on gene expression differences between EP and LP MSC(AT).

Our study is the first to report CD26 as a senescence marker for human MSC(AT). This together with recent findings in umbilical cord MSCs [35], reinforces the relevance of CD26 in MSC senescence. The cell surface location of CD26 led us to develop a new strategy that simultaneously evaluates four MSC(AT) senescence markers. Specifically, a single round of flow cytometry can assess independently cell size, granularity, autofluorescence, and CD26 surface abundance. This approach sets the stage to define in-depth the mechanisms through which MSCs contribute to organismal aging in vivo.

## Conclusions

Our results support the model that MSC(AT) increase *CD26* transcription and CD26 protein levels when undergoing replicative senescence. The CD26^high^ subpopulation is overrepresented in MSC(AT) from adult donors when compared with pediatric donors. This is functionally relevant, as we demonstrate the loss of immunopotency for CD26^high^ MSC(AT). Furthermore, we have developed an approach to prevent the accumulation of senescent MSCs during culture expansion in vitro. It is based on the controlled treatment of proliferating MSCs with senolytics. Taken together, our findings support CD26 surface abundance as a promising marker to assess the quality of MSC(AT) for clinical applications.

## Supplementary Information

**Additional file 1.** Tables (n = 3) and Figures (n = 5).

**Additional file 2.** Excel file with RNA-seq data.

## Data Availability

The datasets used and/or analyzed during the current study are available from the corresponding author on reasonable request.

## Abbreviations

7-AAD: 7‐Aminoactinomycin D
APC: Allophycocyanin
AT: Subcutaneous adipose tissue
cDNA: Complementary DNA
CFSE: Carboxyfluorescein succinimidyl ester
COL4A1: Collagen type IV alpha 1
DAPI: 4′,6-Diamidino-2-phenylindole
DMEM: Dulbecco’s modified Eagle’s medium
DMSO: Dimethyl sulfoxide
DPP4: Dipeptidyl peptidase 4
EP: Early passage
FACS: Fluorescence-activated cell sorting
FBS: Fetal bovine serum
FOXE1: Forkhead box E1
FSC-A: Forward scatter area
gMFI: Geometric mean fluorescence intensity
GSEA: Gene set enrichment analysis
HES1: Hairy and enhancer of split-1
ISCT: International Society for Cell and Gene Therapy
LP: Late passage
MSCs: Mesenchymal stromal cells
MSC(AT): Adipose tissue-derived mesenchymal stromal cells
MSC(M): Bone marrow-derived mesenchymal stromal cells
MSC(WJ): Warton’s jelly mesenchymal stromal cells
PBMCs: Peripheral blood mononuclear cells
PBS: Phosphate buffered saline
qRT-PCR: Quantitative reverse transcription polymerase chain reaction
RNA-Seq: RNA sequencing
RPMI: Rosewell Park Memorial Institute
SA-β-gal: Senescence-associated beta-galactosidase
SASP: Senescence-associated secretory phenotype
SSC-A: Side scatter area
SD: Standard deviation
SPP1: Secreted phosphoprotein 1
WB: Western blot analysis
