# Supplementary material for: CD26 is a senescence marker associated with reduced immunopotency of human adipose tissue-derived multipotent mesenchymal stromal cells
Source: Stem Cell Res Ther. 2022 Jul 26;13:358. doi: 10.1186/s13287-022-03026-4 (PMC9327293; doi:10.1186/s13287-022-03026-4)
Supplement: Supplementary file 1 — Additional file 1. Tables (n = 3) and Figures (n = 5). [file 13287_2022_3026_MOESM1_ESM.pdf]

Supplemental Data

Supplementary Table 1. MSC(AT) Donor Demographics

	**Adult n=7**	**Pediatric n=7**
Age (years, mean ± SD)	68.6 ± 8.1	16.7 ± 2.9
Sex (F)	2	5
Body Mass Index (mean ± SD)	33.0 ± 3.1	24.9 ± 8.0
Diabetes type II	3	-
Hypertension	7	-
Coronary artery disease	6	-

Supplementary Table 2. Gene sets created for Gene Set Enrichment Analysis (GSEA).

**(i) Genes upregulated in cellular senescence**		
AKT1	*IGFBP5*	*RAP1A*
ANGPT1	*IGFBP6*	*RB1*
ANKRD1	*IGFBP7*	*RBL2*
CCL13	*IL13*	*RPE65*
CCL16	*IL15*	*RPS6KB1*
CCL20	*IL1A*	*RXRA*
CCL3	*IL1B*	*S100B*
CCL8	*IL6*	*SAA1*
CDKN1A	*IL7*	*SCN3A*
CDKN2A	*IL8*	*SCUBE3*
CHPF2	*ITGB8*	*SERPINB2*
CLIC3	*JAK2*	*SERPINB7*
COL13A1	*KDELR3*	*SERPINE1*
CXCL1	*KDM6B*	*SLC1A1*
CXCL12	*KRT19*	*SLC9A7*
CXCL2	*KRT34*	*SMARCA4*
CXCL3	*KRTAP1-5*	*SPP1*
CYBB	*L1CAM*	*SSR3*
DKK1	*LMCD1*	*STAT3*
DPP4	*LMNA*	*STMN2*
EEF1E1	*MIF*	*SYNM*
ESM1	*NTN4*	*SYNPO2*
F3	*OXTR*	*TAT*
FOXE1	*PDGFRL*	*TFE3*
FST	*PIM1*	*TGFB1*
GALNT5	*PLAT*	*THBS1*
HAS3	*PLK1*	*TMEM159*
HSP90B1	*PLOD1*	*TMEM59*
ICAM1	*PML*	*TNF*
IFNG	*PPBP*	*TP53*
IGFBP2	*PRNP*	*VCAM1*
IGFBP3	*PSG5*	*WFDC1*
IGFBP4	*PSG9*	

**(ii) Genes downregulated in cellular senescence**	
AMH	*NUPR1*
ANP32B	*OGG1*
APEX1	*P4HA3*
ASF1A	*PARP1*
BMI1	*PHGDH*
BRCA2	*PLXDC2*
CDCA7L	*PLXDC2*
COL6A2	*PODXL*
COMP	*PTGFRN*
CPNE8	*PTMA*
DOK5	*PTPRD*
EFEMP1	*PTPRD*
EZH2	*RB1*
FBL	*RPS27A*
FOXP1	*RUNX1T1*
FXR1	*RUNX2*
GADD45	*SATB2*
GIP1	*SCRG1*
GPAT2	*SERPINF1*
GPAT2	*SGCG*
HGF	*SGCG*
HIF1A	*SIRT1*
HMGB2	*SLC25A37*
KLF4	*SSRP1*
LBR	*TERT*
LTF	*TGFB2*
MCUB	*THBD*
MIR543	*TWIST1*
MIR590	*VEGFA*
MMP1	*XRCC4*
NFIB	*ZMPSTE24*

Gene sets based on previously published data created to validate the presence of a senescence signature in the MSC(AT) samples through GSEA.

Supplementary Table 3. Quantitative RT-PCR gene primer sequences

Gene	Sequence (5'-3')
CDKN2A (p16)	F: GGTCGGGTAGAGGAGGT<br>R: GCGCTGCCCATCATCATG
CDKN1A (p21)	F: TGATTAGCAGCGGAACAAGG<br>R: TGCCAGGAAAGACAACTACTCC
FOXE1	F: GAC CAC GGT GGA CTT CTA CG<br>R: GCG GAC ACG AAC CGA TCT AT
DPP4	F: ATG CCA GGA GGA AGG AAT CT<br>R:TTC CAG GAC TCT CAG CCC TTT
HES1	F: GCT CTG AAG AAA GAT AGC TCG C<br>R: CTC GGT ATT AAC GCC CTC GC
COL4A1	F: CAG GCA CCC CAT CTG TTG AT<br>R: CAT TGC CTT GCA CGT AGA GC
SPP1	F: CTG ATG AAC TGG TCA CTG ATT TTC<br>R: CCG CTT ATA TAA TCT GGA CTG CTT
HPRT	F: GTT GTA GGA TAT GCC CTT GAC TAT<br>R: GAT GTC AAT AGG ACT CCA GAT GTT

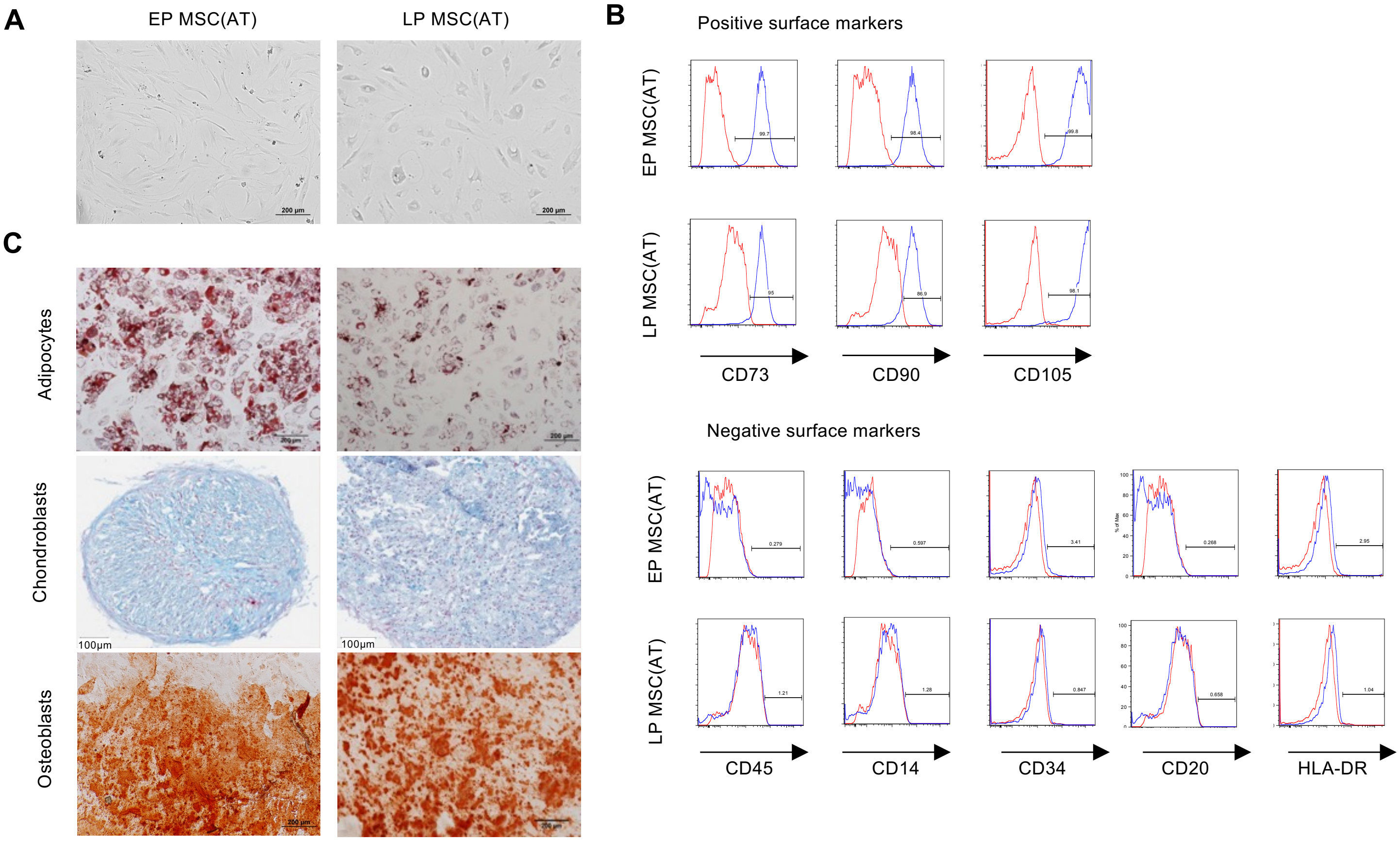

Supplementary Fig. 1. MSC(AT) fulfill the International Society for Cell and Gene Therapy minimal definition criteria. A Representative images of early passage (EP) and late passage (LP) MSC(AT) plastic adherence *in vitro*. **B** EP and LP MSC(AT) express CD73, CD90 and CD105 but lack expression of hematopoietic and activation markers (CD45, CD14, CD34, CD20, HLA-DR). Red and blue histograms represent isotype controls and stained samples respectively. **C** Representative images of EP and LP MSC(AT) tri-lineage in vitro differentiation (i.e. adipocytes, chondroblasts and osteoblasts).

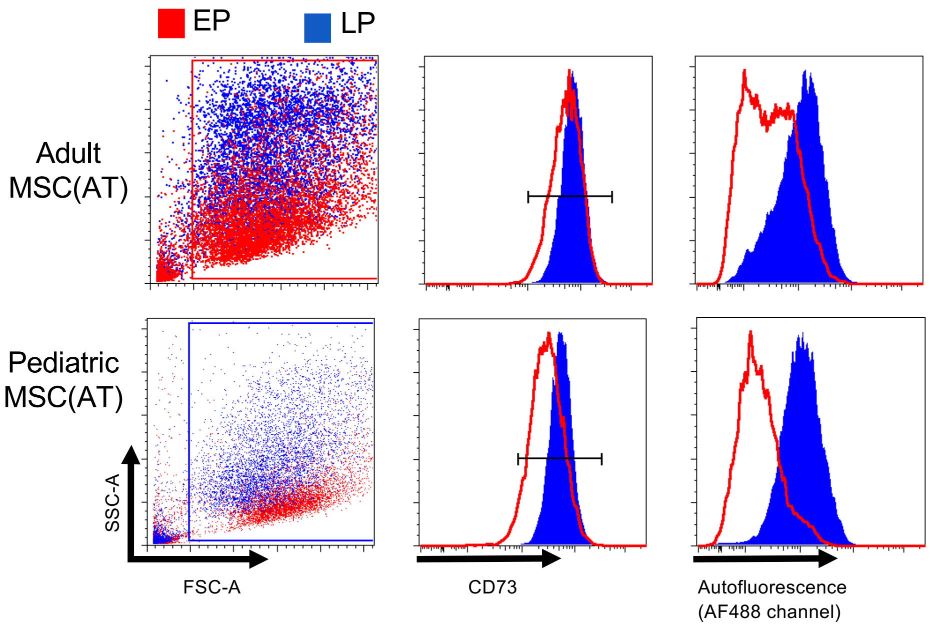

Supplementary Fig. 2. Gating strategy for adult and pediatric MSC(AT) at EP and LP. A merged flow cytometry analysis of EP and LP MSC(AT) from adult and pediatric donors were stained with CD73. Flow cytometry analysis of different cell subpopulations indicates similar surface abundance of CD73 and autofluorescence in LP-MSC(AT).

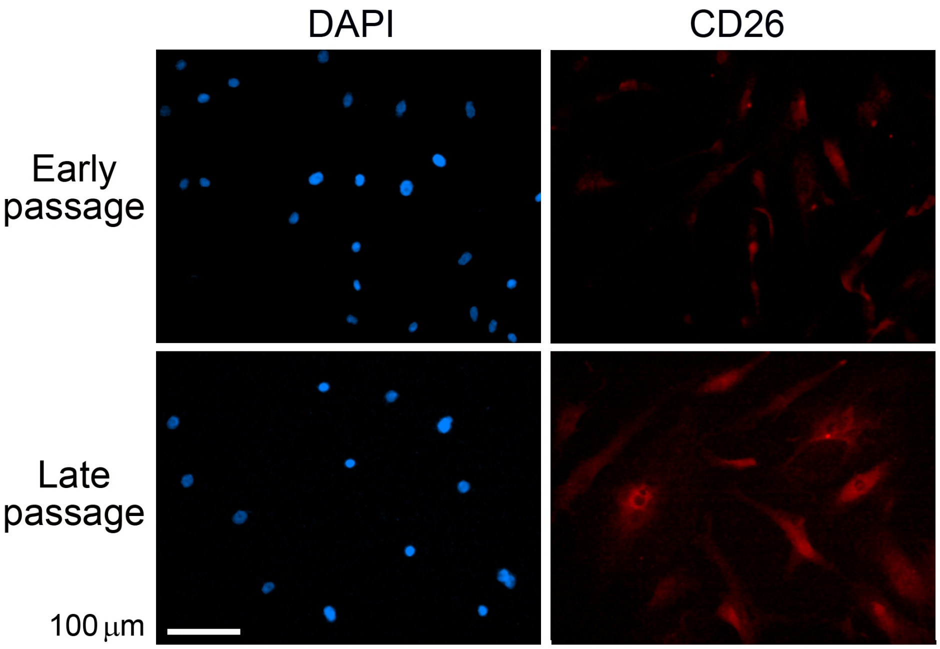

Supplementary Fig. 3. CD26 in early and late passage MSCs (low magnification)

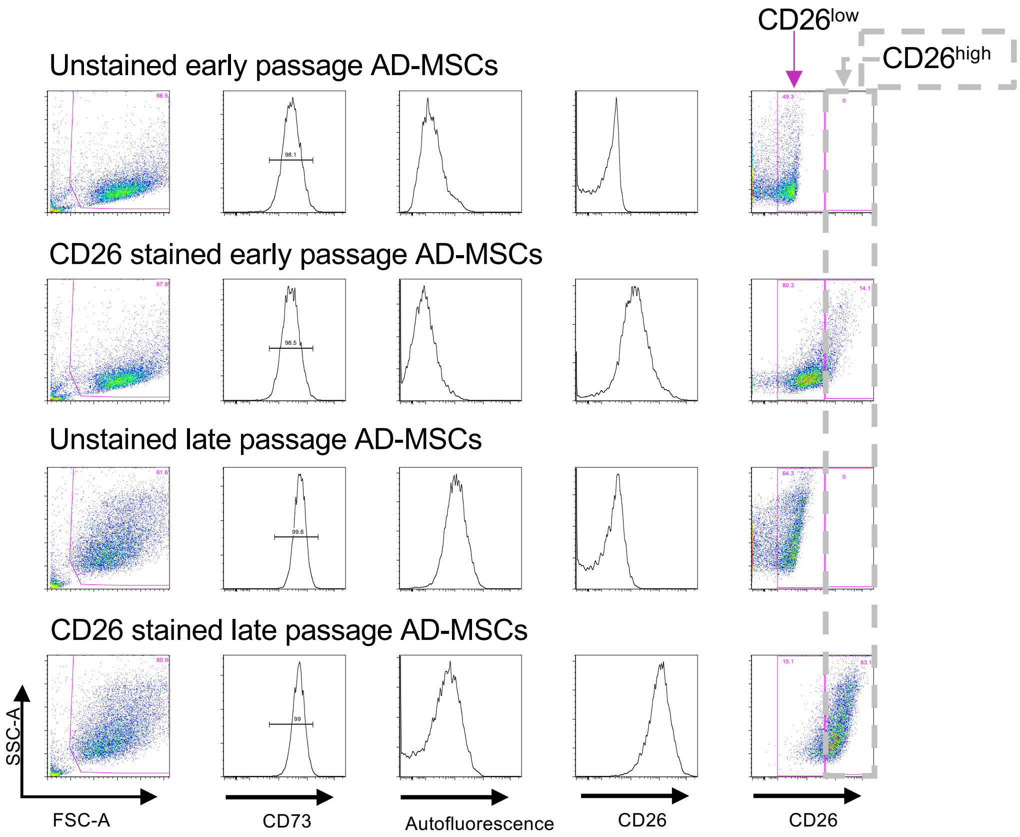

Supplementary Fig. 4. Flow cytometry gating strategy to assess CD26 surface levels in MSC(AT). Columns represent from left to right: gating strategy for cells, $CD73^{+}$ MSC(AT), autofluorescence, and total CD26 surface levels in early and late passage MSC(AT). $CD26^{low}$ and $CD26^{high}$ (gray doted line) gates are shown in the last column. Unstained samples (without CD26 antibody) were used to subtract background fluorescence.

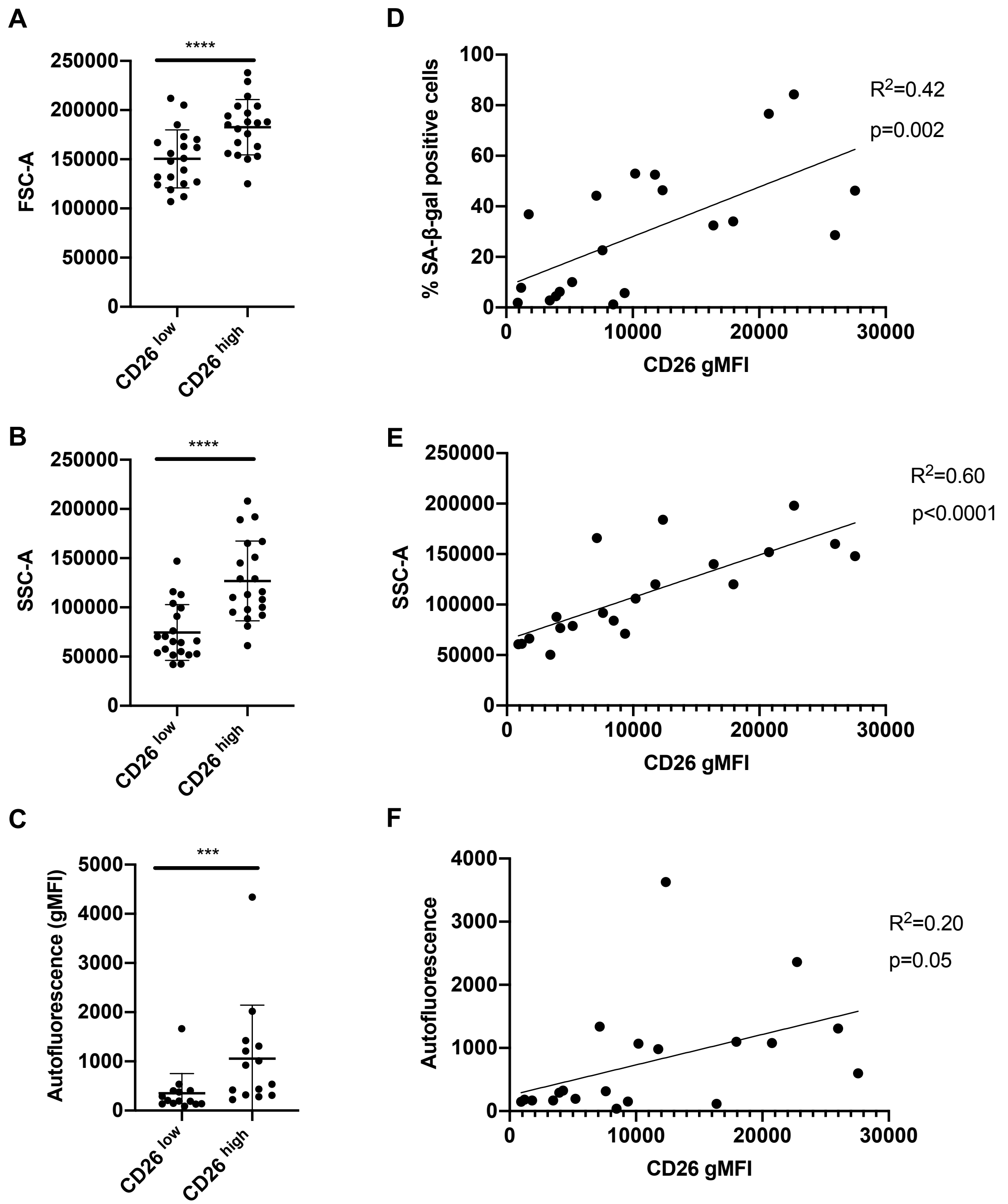

Supplementary Fig. 5. Senescence markers are increased in CD26[high] MSC(AT). Summary graphs of **A** cell size (FSC-A), **B** cell granularity (SSC-A) and **C** autofluorescence in CD26[low] and CD26[high] MSC(AT) populations. n=6 adult and n=4 pediatric MSC(AT) in panels **A** and **B**, while n=4 adult and n=3 pediatric MSC(AT) in panel **C**. Data shown as mean ± SD, comparisons done using paired Wilcoxon tests, ***p<0.001, ****p<0.0001. **D-F** Simple linear regression analyses to correlate CD26 surface levels in early and late passage MSC(AT) with: **D** % SA-β-gal positive cells, **E** cell granularity (SSC-A), **F** autofluorescence. n=6 adult and 4 pediatric MSC(AT), early passage= 4.1±0.6 and late passage= 21.0±6.0.
